# Enhancement of Chemotactic Cell Aggregation by Haptotactic Cell-To-Cell Interaction

**DOI:** 10.1371/journal.pone.0154717

**Published:** 2016-04-29

**Authors:** Tae-goo Kwon, Taeseok Daniel Yang, Kyoung J. Lee

**Affiliations:** Department of Physics, Korea University, Seoul 136-713, Korea; A*STAR Bioinformatics Institute, SINGAPORE

## Abstract

The crawling of biological cell is a complex phenomenon involving various biochemical and mechanical processes. Some of these processes are intrinsic to individual cells, while others pertain to cell-to-cell interactions and to their responses to extrinsically imposed cues. Here, we report an interesting aggregation dynamics of mathematical model cells, when they perform chemotaxis in response to an externally imposed global chemical gradient while they influence each other through a haptotaxis-mediated social interaction, which confers intriguing trail patterns. In the absence of the cell-to-cell interaction, the equilibrium population density profile fits well to that of a simple Keller-Segal population dynamic model, in which a chemotactic current density ${\vec{J}}_{chemo}∼\nabla p$ competes with a normal diffusive current density ${\vec{J}}_{diff}∼\nabla \rho$, where *p* and *ρ* refer to the concentration of chemoattractant and population density, respectively. We find that the cell-to-cell interaction confers a far more compact aggregation resulting in a much higher peak equilibrium cell density. The mathematical model system is applicable to many biological systems such as swarming microglia and neutrophils or accumulating ants towards a localized food source.

## Introduction

Understanding the mechanisms behind cell population dynamics is essential to a wide range of biological processes including development, wound healing, tumor expansion, and immune responses [1–7]. However, it is a very challenging task, since the relevant systems generally involve many different interacting constituents (cells) which are inherently nonlinear and the type of cell-to-cell interactions vary from one case to another. For example, neighboring cells can interact with each other by mechanical forces in a close-packed layer of cells [8, 9]. The local forces can then generate long-range spatio-temporal correlations. Some well-known examples include the ratchet-like tissue movement during dosal closure in the developing *Drosophila* embryo [10] and the waves and swirls in the *in vitro* systems of an expanding epithelial cell sheet [2, 11, 12]. Cells can also be coupled through diffusing chemical agents and matching receptors. One of the most well-studied examples of this type of cell is the traveling-wave chemotaxis of dictyostelium discodium (or dicty) amoebae [13–15]. Briefly, the starvation triggers amoebae to produce and excrete 3’,5’-cyclic adenosine monophosphate (cAMP) that diffuses to the neighboring cells which have cAMP receptors. Not only can the cells amplify the level of cAMP, they can also dissociate cAMP to cGMP in a temporally coordinated manner. Consequently, the cAMP-mediated coupling can bring about large-scale cAMP waves. Therefore, for the case of dicty amoebae, the diffusive coupling and the cell-intrinsic nonlinear kinetics and adaptation are responsible for the collective phenomenon. The amoebae cells also actively move (i. e. chemotaxis) towards the higher concentration of cAMP while experiencing the positive slopes of cAMP waves.

In some cases, the chemical agents released by crawling cells do not diffuse but stay behind the cells, and become encapsuled into small vesicles (typical size, 40∼100 nm in diameter) called as exsosomes. Alternatively, they become bound to the two-dimensional substrate (or three-dimensional matrix) on which the cells are placed [16–18]. In our recent work, we showed that in cultured microglia, the immune cells of the brain [19], a number of chemical markers (e.g. *α*5-integrin) are left behind the crawling traces of the cells and that these non-diffusing (but degrading) markers serve as intercellular signaling agents guiding their motility [20], enabling the cells to form intricate evolving network of trails [21]. In short, microglia exhibit “haptotaxis” by following the trails they generate. Generally speaking, “haptotaxis” refers to the directed movement of cells controlled by the relative strengths of peripheral adhesions into some substrates.

Microglia also exhibit “chemotaxis” in response to the concentration gradient of adenosine monophosphate (ATP) [22, 23]. “Chemotaxis” is the directed movement of bacteria, eukaryotic cells, or multi-cellular organisms toward concentrations of environmental chemoattractants. Therefore, microglia movements can be driven by several different agents. Multi-modal communications are indeed very common for biological cells. Neutrophils rely on integrins and lipid leukotriene B4 (LTB4) for swarming [24]; they also release exsosomes behind their moving trails as for intercellular signaling [16]. Numerous chemokines are known to be involved in chemotaxing tumor cells [7] and tumor cell migration is driven by haptotaxis as well as chemotaxis [25]. Indeed, for many realistic biological settings, cell-to-cell interactions are mediated by several different modalities with many different intercellular signaling molecules working together. However, the potential interplay between these different modalities is largely unexplored.

This paper, in particular, investigates the role of a haptotaxis-mediated cell-to-cell interaction in the aggregation dynamics of the chemotaxing of the cells in response to a globally imposed chemoattractant gradient. The paper is based on the computer-simulations of a population of model cells having some key features of crawling microglia in *in vitro* culture. More specifically, this work is motivated by our recent experimental observations concerning microglia trail network formation and the chemotactic behavior of microglia. While the haptotaxis-mediated trail network formation and the chemotactic responses of microglia to ATP have been studied separately in the past, their interplay has not yet been addressed. Thus, this paper may be considered as an exposition of the ways in which such an interplay can be interesting and useful. Most importantly, we find that the haptotaxis-mediated trail network can greatly enhance the aggregation of chemotaxing cells.

## Results and Discussion

### Properties of the rule-based mathematical model cell mimicking a microglial cell

**Fig 1:**
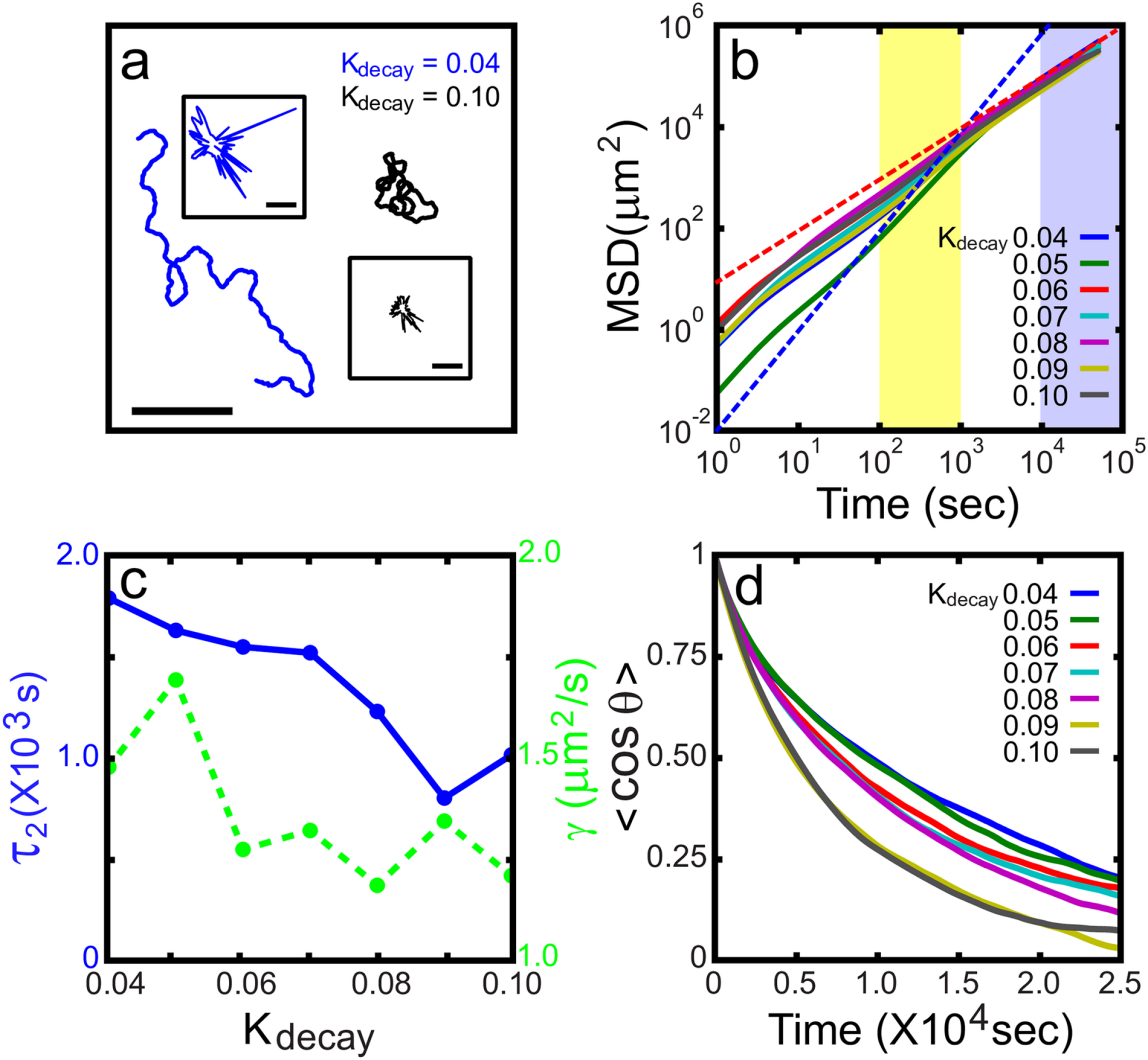
Directional persistence of a mathematical model cell. (a) Two sample traces of crawling model cells. (b) Mean squared displacement for seven different values of control parameter *K*_*decay*_. (c) Auto-correlation functions of the instantaneous direction of crawling. (d) Directional persistent time *τ*_2_ and superdiffusiveness exponent *γ* vs. *K*_*decay*_. The insets in (a) show two representative snapshot images of the model cells. The blue (red) dashed line in (b) has a slope of 2 (1). The large (small) scale bar is 250 (20) *μm*. The variations in *τ*_2_ and *γ* brought about by a different initial condition (positions and random seed number) are less than 0.5% (n = 5).

The model cell used in this study is essentially the same as that originally proposed by Satulovsky *et al*. [26]. Basically, the cell is a simply-closed loop moving on a flat two-dimensional surface (see Fig 1a). The movement of the loop is determined by two scalar fields: the activator $S^{+}\left(\vec{r},t\right)$ and the inhibitor *S*^−^(*t*). $S^{+}\left(\vec{r},t\right)$ depends on the position $\vec{r}$ (the position vector along the cell perimeter with respect to the centroid of the cell) as well as time, whereas *S*^−^(*t*) is a global variable that only depends on the time. At every iteration time step, $\vec{r}$ (along the perimeter) can either advance, retreat or stay based on the following set of rules. Retraction occurs when $S^{+}\left(\vec{r},t\right)\leq S^{-}\left(t\right)$ and the rate of retraction is governed by the following equation:

$$\begin{array}{c} \partial |\vec{r}|/\partial t=-max([|\vec{r}|-r_{min}]R^{-},0), \end{array}$$ (1)

where *r*_*min*_ is the constant minimum radius and *R*^−^ is the retraction rate constant. The function *max*(*x*, *y*) selects the larger value of *x* and *y*. Protrusion occurs when $S^{+}\left(\vec{r},t\right)>S^{-}\left(t\right)$ at a rate governed by the following equation:

$$\begin{array}{c} \partial |\vec{r}|/\partial t=max\left(G\left(R^{+}\right),0\right), \end{array}$$ (2)

where *R*^+^ is the average protrusion rate and *G*(*R*^+^) is a random number generated from a Gaussian distribution with the mean *R*^+^ and variance *R*^+^.

The activator $S^{+}\left(\vec{r},t\right)$ evolves according to the following equation:

$$\begin{array}{c} \begin{array}{c} \partial S^{+}\left(\vec{r},t\right)/\partial t=K_{diff}\nabla^{2}S^{+}\left(\vec{r},t\right)-K_{decay}S^{+}\left(\vec{r},t\right)\\ +max[G\left(f\left(S^{+}\left(\vec{r},t\right)-S^{-}\left(t\right),\gamma ,\lambda +P_{baseline}\right)N_{burst}\right),0]. \end{array} \end{array}$$ (3)

The first term accounts for the diffusion of *S*^+^ along the cell perimeter and the second term renders a self-decomposition of *S*^+^. The last term is a stochastic positive feedback loop accounting for both the local stimulation and the existence of a random signal. The function *f*(*x*, *γ*, *λ*) = 0 for *x* < *λ* and (*x* − *λ*) for *x* ≥ *λ*, where *λ* is a threshold value for the feedback. *P*_*baseline*_ refers to the rate of random bursts caused by the internal baseline activities. The function *G* again represents a random number generated from a Gaussian distribution.

The retraction signal is governed by the global inhibition rule $S^{-}\left(t\right)=C^{-}A\int S^{+}\left(\vec{r},t\right)d\vec{r}$, where *C*^−^ is the inhibition constant, *A* is the total area of the cell, and the integration is a line integral over the entire cell border, which is composed of 360 pixels (i.e. 1 pixel for 1 degree with respect to the centroid). Each pixel corresponds to 0.286 *μ*m and one iteration time step is 1 s. At each iterative time step, the formation of focal adhesions and their detachments are assigned stochastically to the points along the cell perimeter with a probability $P_{fa}^{+}$ and $P_{fa}^{-}$, respectively. Retraction is inhibited when a perimeter point hits a focal adhesion. Again, all the rationales for the kinetics and mechanics are fully described in the original paper by Satulovsky et al. [26].

It is known that the rule-based phenomenological model can successfully recapitulate many different types of cell morphologies and motilities, mimicking cells such as keratocytes, neurons, and amoebae. In the current study, the following set of parameter values is chosen to match some of the important characteristics of a typical microglia: *R*^+^ = 0.103 *μ*m/s, *R*^−^ = 0.0281 1/s, *K*_*diff*_ = 11.9 *μm*^2^/s, *N*_*burst*_ = 13, *P*_*baseline*_ = 0.181 1/(s· *μ*m), *λ* = 3.22 1/*μ*m, *γ* = 29.1 1/s, *C*^−^ = 1.93 × 10^−5^ 1/*μ*m^3^, $P_{fa}^{+}=0.0003$, $P_{fa}^{-}=0.0058$, and *r*_*min*_ = 2.857 *μ*m. Only the parameter *K*_*decay*_ is varied as a control parameter.

Fig 1a shows two exemplary passages traced out by two freely crawling model cells having different values of *K*_*decay*_. As clearly illustrated in Fig 1b, for the time domain ranging from hundreds to a few thousand seconds (yellow shaded area in Fig 1b) the cells are superdiffusive with the exponent *γ* of the mean squared displacement <*R*^2^(*t*)> ∼ *t*^*γ*^ with the range of 1 < *γ* < 2 (see Fig 1c, green dots). Overall, *γ* decreases as *K*_*decay*_ increases, and this tendency is reflected well in the two exemplary traces shown in Fig 1a. The directional persistence of a given passage can also be estimated from < cos*θ*(*t*) > vs. *t*, where *θ*(*t*) is the angle between two tangent vectors obtained at two different locations along the passage that are separated by time *t* (see Fig 1d): < cos*θ*(*t*) > is fitted to a function $Ae^{-t/\tau_{1}}+\left(1-A\right)e^{-t/\tau_{2}}$; and then *τ*_2_ is then considered as the persistent time of crawling, while *τ*_1_ is an estimate of the small time interval (typically, 3∼7 minutes) between two successive small-angle zigzag turns (see Ref. [27] for details). Similar to the superdiffusiveness exponent *γ*, overall the persistent time *τ*_2_ is also a decreasing function of *K*_*decay*_, as shown in Fig 1c.

### Chemotaxing model cell population under a globally imposed concentration gradient of an attractant

Motivated by the earlier experimental studies showing chemotactic behaviors of microglia towards a high concentration of ATP [22, 23], we modified the single cell model to be responsive to an extrinsically imposed chemotactic drive; specifically, we added a new term $p(\vec{r^{\prime }})$ to Eq (3), where $\vec{r^{\prime }}$ represents a two-dimensional position vector with respect to a laboratory frame and $p(\vec{r^{\prime }})$ is a concentration map of the chemoattractant. The modified equation for *S*^+^ reads,

$$\begin{array}{c} \begin{array}{c} \partial S^{+}\left(\vec{r},t\right)/\partial t=K_{diff}\nabla^{2}S^{+}\left(\vec{r},t\right)-K_{decay}S^{+}\left(\vec{r},t\right)\\ +max\left[G\left(f\left(S^{+}\left(\vec{r},t\right)-S^{-}\left(t\right),\gamma ,\lambda +P_{baseline}\right)N_{burst}\right),0\right]+p\left(\vec{r^{\prime }}\right). \end{array} \end{array}$$ (4)

As the level of *p* increases, the production of activator *S*^+^ also increases. Consequently, the cell boundary facing upwards of the gradient (i. e. higher value of *p*) is more likely to have a higher value of *S*^+^ than that of the cell boundary facing downwards of the gradient. On the other hand, *S*^−^ is a global variable depending on the spatial average of *S*^+^. Therefore, it will not be greatly affected by the *p* gradient. Subsequently, the cells are more likely to move up the gradient on the average.

**Fig 2:**
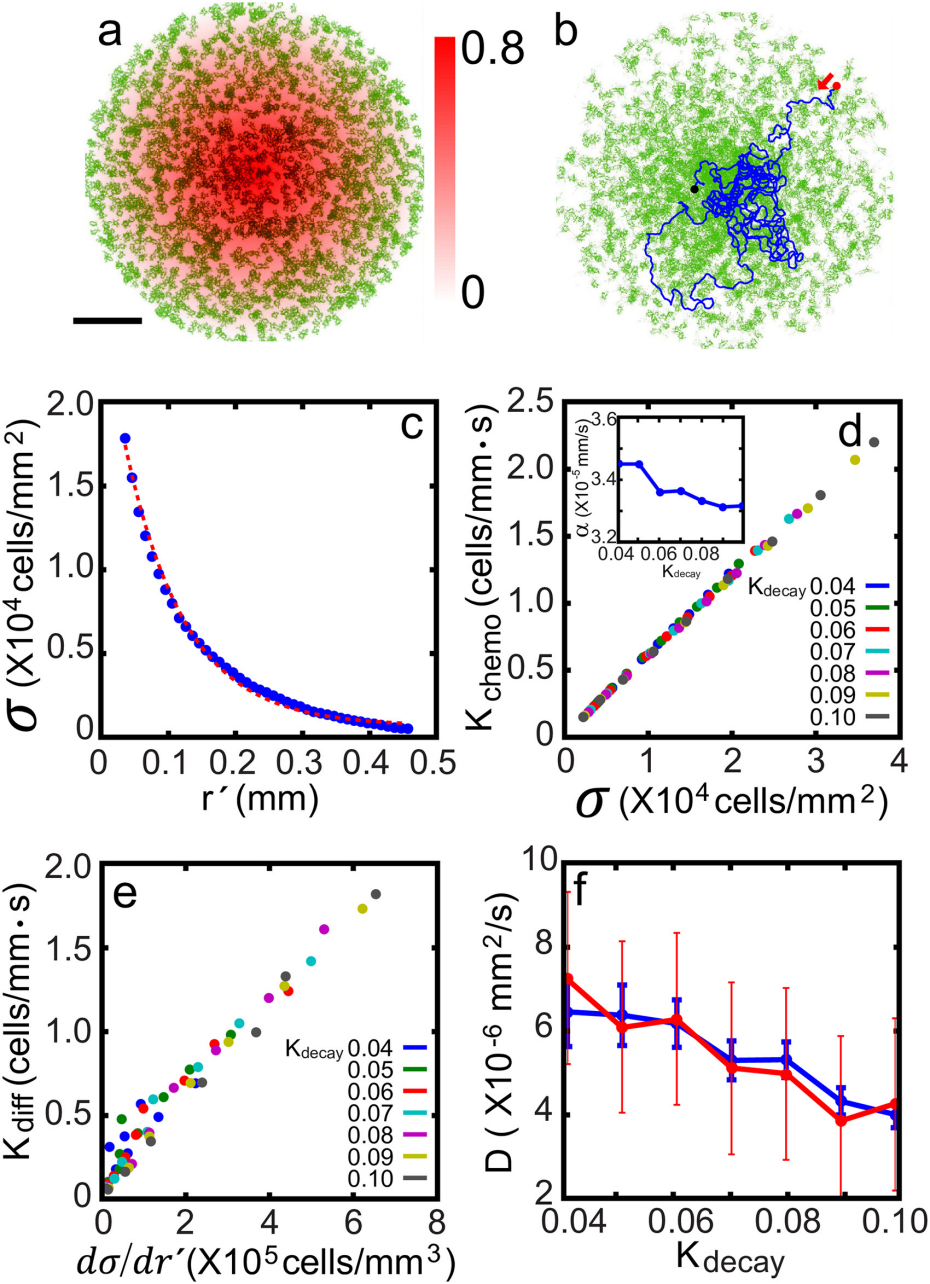
Chemotaxing model cell population. (a) 2,000 model cells (green) under a constant radial gradient of chemoattractant *p* (red). (b) Equilibrium density state for *K*_*decay*_ = 0.04 superimposed with an exemplary trace (blue) of a cell (red: start, black: end). (c) Density distribution at an equilibrium. (d) Chemotactic surface current density vs. surface density; the inset plots the slopes. (e) Diffusive surface current density vs. surface density gradient. (f) Effective diffusion coefficients [blue: estimation from (e), red: estimation from Fig 1b]. Blue (red) error bars in (f) represent the uncertainty associated with the linear least square fitting of the data in Fig 2e (Fig 1b). The red dashed line in (c) represents an exponential fit of the data. The variations in *α* and *D* brought about by a different initial condition are less than 0.5% (n = 5).

In Fig 2a, we placed 2,000 non-interacting model cells uniformly but randomly, and imposed a conically shaped chemoattractant $p(\vec{r^{\prime }})$ concentration image [the opening angle of the cone, *θ* = 2 arctan (1.25*r*′)] to the system to investigate its subsequent chemotactic response. For the given *p* gradient, although their long-term motilities are biased towards the peak of *p* on average, the individual model cells still trace out quite random passages locally due to their built-in stochastic elements: An exemplary trace is given in Fig 2b. In the long run, the population cell density *σ* equilibrates with a peak in the middle and it fits fairly well to an exponential function as shown in Fig 2c (see S1 Movie). To better understand the origin of this equilibrium profile, we measured the chemotactic (surface) current density *K*_*chemo*_ as a function of *σ* and the diffusive current density *K*_*diff*_ as a function of $\frac{\partial \sigma }{\partial r^{\prime }}$ for several different values of *K*_*decay*_ as shown in Fig 2d and 2e, respectively. Here, *K*_*chemo*_ (or *K*_*diff*_) is measured to be the number of cells, moving up (or down) the *p*-gradient, crossing a unit arc-length along a circle about the peak per unit time.

The measured *K*_*chemo*_ is a linear function of *σ*. This seems quite natural, because the cells are essentially identical; thus, they have the same crawling tendency towards the peak of *p* and for the given setting there is no cell-to-cell interaction. Therefore, *K*_*chemo*_ should be a linear function of *σ*. Indeed, the linear form *K*_*chemo*_ = *ασ* (with *α* being the chemotaxis strength) has been implemented widely as the simplest form of chemotactic current density [28]. The chemotatic strength *α*, which can also be viewed as a net chemotatic velocity, is a slowly decaying function of *K*_*decay*_ (see the inset of Fig 2d). Considering that the model cells are built around nonlinear kinetics, rule-based nonlinear mechanics and several stochastic features, the nearly linear relationship cannot be explained easily. Surely, *α* will depend on many different factors such as instantaneous crawling speed, directional persistence in crawling, angular diffusivity, and memory.

On the other hand, *K*_*diff*_ is measured to be a linear function of the density gradient $\frac{\partial \sigma }{\partial r^{\prime }}$ as shown in Fig 2d. The slope of *K*_*diff*_ vs $\frac{\partial \sigma }{\partial r^{\prime }}$ is nothing but effective diffusion coefficient *D*; it is evaluated for several different values of *K*_*decay*_ and is shown in Fig 2f (blue line). In other words, although the individual model cells are superdiffusive and have a directional persistence for the intermediate time range (shaded in yellow in Fig 1b), for the long time domain (*t* ≳ 10,000 s, shaded in violet in Fig 1b), which is relevant for establishing the density equilibrium state, they can be viewed simply as a Brownian particle following the normal Fick’s diffusion law. In fact, similar values of *D* are also obtained by fitting the long time domain portions (10,000 < *t* < 20,000 s) of the MSD curves of Fig 1b to a straight line with slope 1 as shown in Fig 2f (red line). Within the estimated error bars associated with the linear least-square fitting, the two different estimations agree fairly closely. *D* is measured to be a decreasing function of *K*_*decay*_ or an increasing function of the directional crawling persistence. Within the long time regime, in which the cells can be viewed simply as a random-walker, a decreased directional persistence in the crawling cell is effectively similar to having a smaller step size for a random walker. Besides, we found that the angular diffusivity increases as *K*_*decay*_ increases (not shown, see Ref. [27]). Consequently, parameter *K*_*decay*_ strongly influences *D* and Fig 1a also clearly illustrates this point.

### Trail formation by haptotaxing model cell population

**Fig 3:**
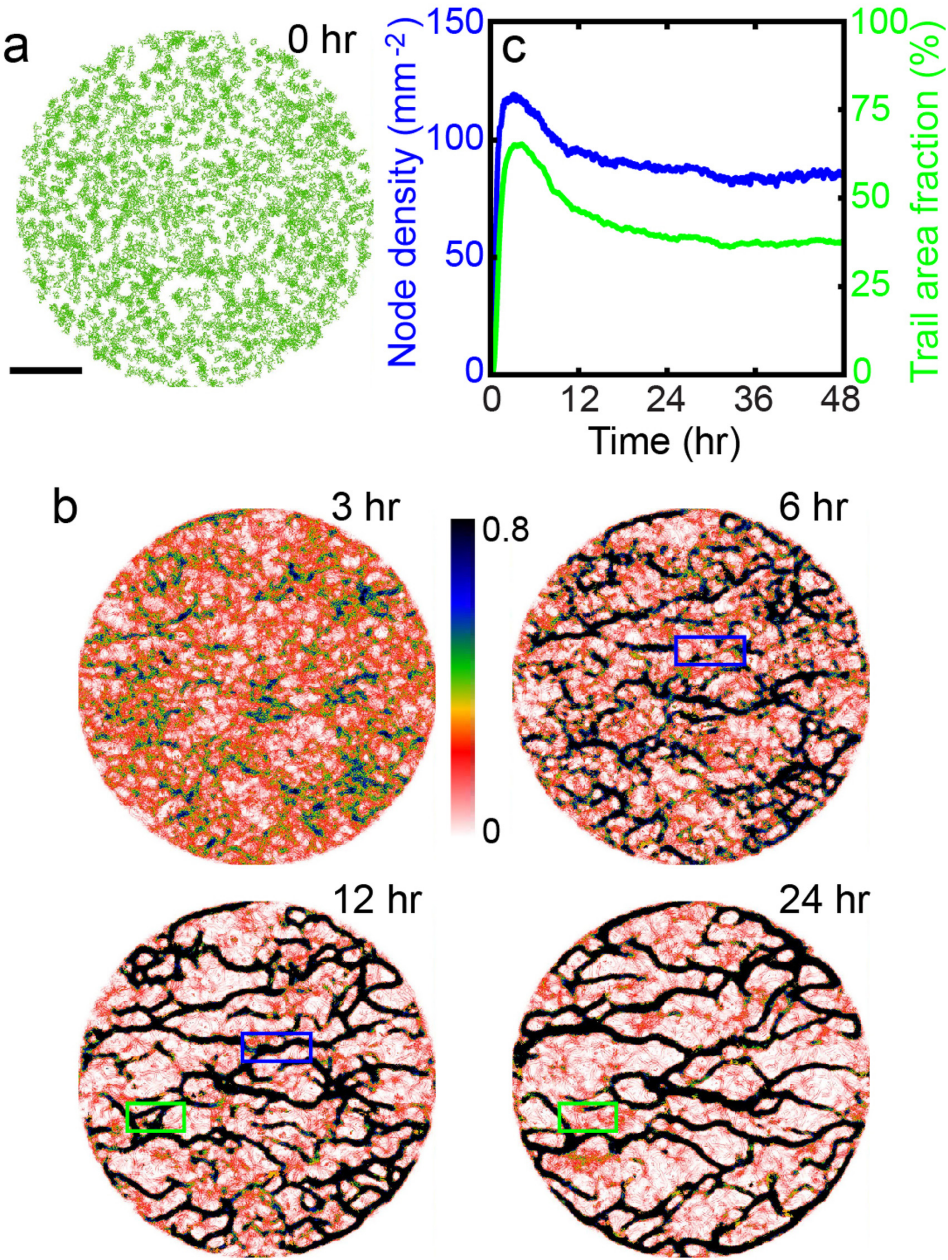
Trail network formation in a population of interacting model cells. (a) Initial state showing 2,000 cells (*K*_*decay*_ = 0.04), (b) Sequence of pseudo-color images of *q* attractant showing the development of a network of trails, and (c) Node (i. e. trail junctions) density and the areal percentage of trails vs. time. Even for the long-term steady state, the network is slowly but constantly evolving with the formation of new trails and the decay of rarely visited portions of trails. The pair of blue (green) boxes in (b) illustrates a case of an emerging (decaying) trail in time.

We previously showed that crawling microglia can form intricate networks of trails (see S1 Fig) and the mathematical model cells can faithfully recapitulate many of the key features of microglial trail networks if they are coupled by a haptotaxis-mediated coupling [21]. Here, we reproduced the phenomenon in a disk geometry (see Fig 3). For the haptotaxis-mediated coupling, we added a chemoattractant

$$\begin{array}{c} q\left(\vec{r^{\prime }},t\right)=q_{0}\sum_{i}e^{-\left(t-t_{i}\right)/\tau_{decay}} \end{array}$$ (5)

to Eq (3). $q(\vec{r^{\prime }},t)$ is a scalar field representing the concentration of a number of proteins produced and deposited at $\vec{r^{\prime }}$ by crawling cells. *t*_*i*_ represents the specific time of *i*th visit to the position $\vec{r^{\prime }}$ previously made by one of the cells. After being deposited, the attractant self-decomposes with a time constant *τ*_*decay*_. From the viewpoint of *S*^+^ kinetics, the role of *q* is essentially identical to that of the chemoattractant *p*: When a model cell encounters a *q*-deposited trail, it will most likely be guided along the trail since *q* is a *S*^+^ enhancing chemical agent similar to *p*. The scalar field *q* is, however, a dynamic variable that evolves spatio-temporally, while *p* represents the extrinsically imposed fixed landscape of a chemoattractant.

Again, from an initially uniform, randomly-distributed state (Fig 3a), the model cells are allowed to move and release *q* behind their passages. As they crawl around and recognize their neighboring *q*-landscape, their motile behaviors become biased to follow the nearby “foot prints” created by other cells. Consequently, an evolving network of trails is formed, as illustrated in Fig 3b. For the chosen set of parameter values, small trails initially emerge, forming a dense fuzzy network (Fig 3b, t = 3 hr), which eventually becomes a more refined network of trails (Fig 3b, t = 24 hr, also see S2 Movie). The network evolves constantly as new branches form, while some less visited portions disappear (compare the two pairs of boxed areas in Fig 3b). From a uniformly distributed state, the establishment of a mature network typically takes about 24 hrs, as shown in Fig 3c, in which the trails are defined to be the area with *q* > 0.22. Subsequently, the trails are “skeletonized” with an open source program *ImageJ* and each crossing junction is identified as a node.

**Fig 4:**
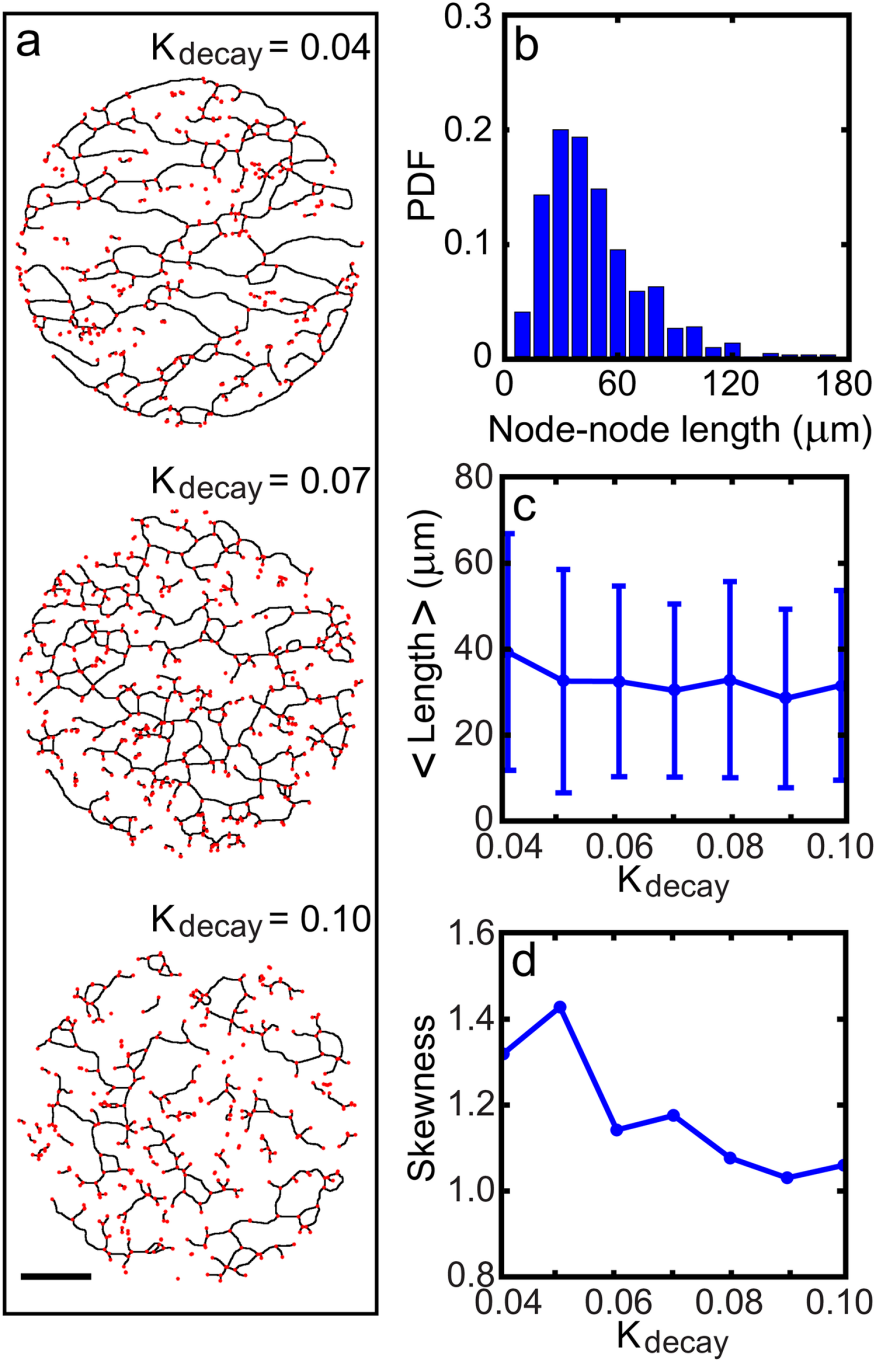
Shapes and statistical properties of the trail networks for different values of *K*_*decay*_. (a) Skeletonized networks of trails (red stars mark the nodes). (b) Probability density function of the distance between two immediately connected neighboring nodes. (c) Mean distance vs. *K*_*decay*_ (error bars represent standard deviations). (d) Skewness vs. *K*_*decay*_. The standard deviation of the mean distance and and that of the skewness are about 10% with different initial conditions (n = 5).

Fig 4a depicts various skeletonized trail networks for different values of *K*_*decay*_. Again, for the range of parameter values we have explored, as the value of *K*_*decay*_ decreases, the directional persistence in crawling improves and subsequently the trail network becomes coarser and more percolated. A typical histogram of inter-node-distance (or “edge”) is given in Fig 4b. Although the average values and standard deviations do not change significantly for different values of *K*_*decay*_ (see Fig 4c), the skewness changes significantly as expected (see Fig 4d). The mean instantaneous crawling speed along the trails is almost a linearly decreasing function of *K*_*decay*_, ranging approximately from 5.7 to 6.7 *μ*m/min: As the trail becomes straighter, the speed at which the cells move increases. However, the speed at which the model cells crawl is about 30% faster in the absence of any *q*-trails (see S2 Fig).

### Effective aggregation assisted by cell-to-cell interaction resulting trail network

**Fig 5:**
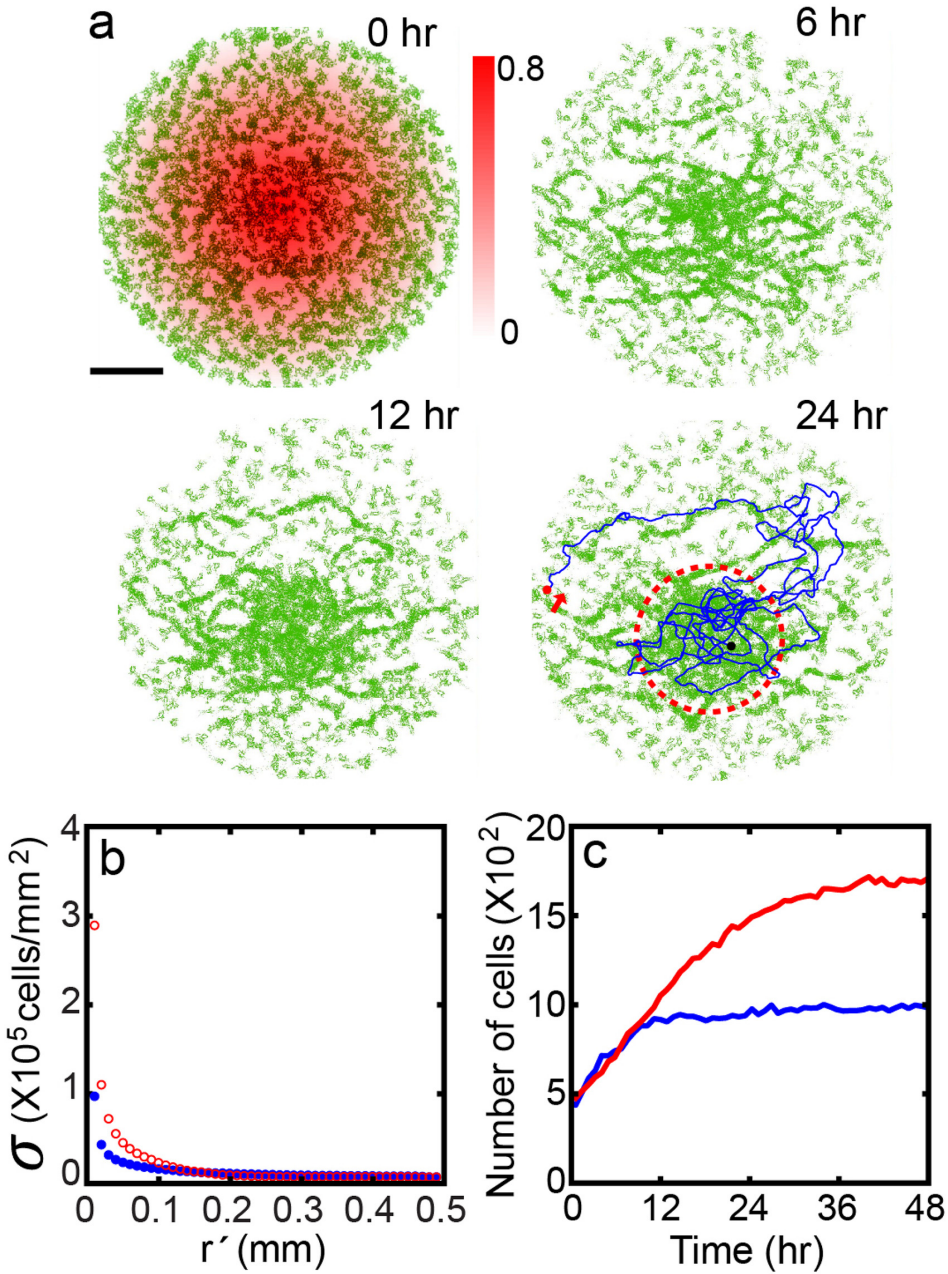
Evolution of a population of chemotaxing cells with haptotactic cell-to-cell interactions. (a) Sequence of snapshot images of a model cell population (*K*_*decay*_ = 0.04). (b) Cell density vs. radial position from the centroid of the population and (c) Number of cells within a circle of radius 200*μm* from the centroid (e.g. the circle with a dashed line at 24 hr [blue: chemotaxis only, red: both chemotaxis and haptotaxis]. The blue line for the 24 hr in (a) is an exemplary trace of a cell.

Finally, we question the consequence of implementing the haptotactic cell-to-cell interaction for the cells that are already chemotaxing. The cells experience the same static *p*-gradient as shown in Fig 3a (see Fig 5a, t = 0), but they now also have the *q*-mediated interaction forming trails. Due to the chemotactic force of the *p*-gradient, initially the self-organized trails exhibit some tendency to orient themselves towards the center (see Fig 5a, t = 6 hr, also see S3 Movie). On the other hand, the presence of the trail network can break the circular symmetry of the system and render the centroid of the “high density core area” not in the middle of, but off-center to, the peak of *p*. The core-centric chemotactic force competes with the non-centric haptotactic guide imposed by the trails. For the chosen set of parameter values and the *p*-landscape, we find that the high density core centroid is typically 32%∼35% off-centered while its azimuthal location changes with a different initial state of the cell population (see S3a Fig). Importantly, however, the position of the (time-averaged) maximum cell density is still located at the peak of *p* (see S3b Fig), which is also the global maximum of *p* + *q* (see S3c Fig).

Fig 5b compares two equilibrium density profiles as a function of the radial distance from the peak of the population density, which is also the center of the disk domain: one corresponds to the case having the *q*-mediated haptotactic interaction (red line) and the other corresponds to the case without it (blue line). Clearly, the cells are aggregated more around the peak (*r*′ = 0) for the case with the *q*-mediated social interaction. In fact, in equilibrium, the number of cells in the core area (bounded by the dashed circle in Fig 5a 24hr) is almost two times that of the case with no *q*-mediated interaction (see Fig 5c). In other words, the existence of trails markedly improves the cell gathering in the core area. This can be attributed to the structure of the trail network (see S3b Fig for example) that encircles the peak core area. The structure effectively reduces the diffusive flux down the hill, guiding the cells along the azimuthal direction. On the other hand, the chemotactic uphill flux is not so much affected by the existence of the trails as the cells channels through the trails towards the peak. In equilibrium, the majority of cells are being trapped within the core area, where the level of *p* + *q* is very high (see S3c Fig).

## Conclusions

We conducted numerical simulations of the population dynamics of mathematical model cells that have a built-in directional persistence in their crawling for three different cases: 1) when they perform chemotaxis following an externally imposed gradient of a chemical attractant, 2) when there is a haptotaxis-mediated cell-to-cell social interaction, and finally 3) when both are present simultaneously. Originally, the model system was developed to understand and mimic the properties of microglia that exhibit both chemotaxis and haptotaxis. However, while it seems that all cell movements are generally driven by several different factors and mechanisms working simultaneously, very little has been discussed so far about their collaborative interplays. This work may be considered as an exposition of the way in which such an interplay can be synergistically useful or important in biology. Most significantly, we demonstrated that a haptotaxis-mediated cell-to-cell interaction could significantly enhance the chemotactic aggregation of cells towards the area of maximum concentration of a globally imposed attractant.

Several other results were also notable. For the case of 1) (chemotaxis only), the steady-state cell density profile fits rather well to that of the simple Keller-Segel chemotaxis model [28], in which a chemotactic flux *J*_*chemo*_ = *αρ* competes with a diffusive flux *J*_*diff*_ = *D*∇*ρ* to result in a density profile of an exponential function. For the slow time scale which is relevant for reaching an equilibrium density profile, the individual model cells can be viewed simply as a normal Brownian particle. Considering that no cell-to-cell interaction occurs, the linear dependence of *J*_*chemo*_ on *ρ* seems natural. It is also worth noting that in the case of chemotaxis with a haptotactic social interaction, the aggregation core (i.e. the centroid of the population) is in general not centered on the peak of the chemoattractant *p* profile and the overall azimuthal symmetry is broken. Therefore, a symmetry-breaking instability seems to be responsible for producing a localized density structure on the trail network by a self-enhancing feedback mechanism. This needs to be further explored.

## Supporting Information

S1 Fig Networks of trails formed by crawling rat microglia:(left) snapshot image, (right) superimposed image. Cell density = 200 cells/mm^2^ in (a) and 1,000 cells/mm^2^ in (b). (50 hr duration of) 6,000 successive phase-contrast snapshot images taken at every 30 seconds (beginning from 3 days in vitro after the initial seeding) are superimposed to reveal the existence of trail patterns on the second column.
(TIF)

S2 Fig Mean instantaneous crawling speeds for the model cells under two different conditions. The speeds are estimated based on a time interval of 60 s.
(TIF)

S3 Fig Localized density states formed by chemo- and hapto-taxing population of model cells. (a) Four different steady states for four different initial seeding conditions. (b) Time-accumulated (48 hr) cell count map of the case shown in the top-left frame in (a). (c) Snapshot image of *p* + *q* concentration map of the steady state shown in (b). The cell count map shown in (b) is based on a two-dimensional array of a small disk (of radius 10 pixels or 2.86 *μ*m) windows.
(TIF)

S1 Movie Model cell population (*n* = 2,000, *K*_*decay*_ = 0.04) chemotaxing towards the peak (center) of *p*-landscape. (MOV)

S2 Movie Trail network formation in a population of interacting (*n* = 2,000, *K*_*decay*_ = 0.04) model cells in the absence of *p*-gradient. (MOV)

S3 Movie Trail network formation in a population of interacting (*n* = 2,000, *K*_*decay*_ = 0.04) model cells in the presence of *p*-gradient. (MOV)

## References

[1] Dombrowski C, Cisneros L, Chatkaew S, Goldstein RE, Kessler JO. Self-concentration and large-scale coherence in bacterial dynamics. Phys Rev Lett. 2004;93:098103 10.1103/PhysRevLett.93.098103 15447144

[2] Poujade M, Grasland-Mongrain E, Hertzog A, Jouanneau J, Chavrier P, Ladoux B, et al Collective migration of an epithelial monolayer in response to a model wound. Proc Natl Acad Sci USA. 2007;104:15988–93. 10.1073/pnas.0705062104 17905871 PMC2042149

[3] Chtanova T, Schaeffer M, Han SJ, van Dooren GG, Nollmann M, Herzmark P, et al Dynamics of neutrophil migration in lymph nodes during infection. Immunity. 2008;29:487–96. 10.1016/j.immuni.2008.07.012 18718768 PMC2569002

[4] Bonner JT. The social amoebae: the biology of cellular slime molds. Princeton: Princeton University Press; 2008.

[5] Weijer CJ. Collective cell migration in development. J Cell Sci. 2009;122:3215–23. 10.1242/jcs.036517 19726631

[6] Angelini TE, Hannezo E, Trepat X, Fredberg JJ, Weitz DA. Cell Migration Driven by Cooperative Substrate Deformation Patterns. Phys Rev Lett. 2010;104:168104 10.1103/PhysRevLett.104.168104 20482085 PMC3947506

[7] Roussos ET, Condeelis JS, Patsialou A. Chemotaxis in cancer. Nat Rev Cancer. 2011;11:573–87. 10.1038/nrc3078 21779009 PMC4030706

[8] Shraiman BI. Mechanical feedback as a possible regulator of tissue growth. Proc Natl Acad Sci USA. 2005;102:3318–23. 10.1073/pnas.0404782102 15728365 PMC552900

[9] Puliafito A, Hufnagel L, Neveu P, Streichan S, Sigal A, Fygenson DK, et al Collective and single cell behavior in epithelial contact inhibition. Proc Natl Acad Sci USA. 2011;109:739–44. 10.1073/pnas.1007809109 PMC3271933 22228306

[10] Solon J, Kaya-Copur A, Colombelli J, Brunner D. Pulsed forces timed by a ratchet-like mechanism drive directed tissue movement during dorsal closure. Cell. 2009;137:1331–42. 10.1016/j.cell.2009.03.050 19563762

[11] Trepat X, Wasserman MR, Angelini TE, Millet E, Weitz DA, Butler JP, et al Physical forces during collective cell migration. Nat Phys 2009;5:426–30. 10.1038/nphys1269

[12] Serra-Picamal X, Conte V, Vincent R, Anon E, Tambe DT, Bazellieres E, et al Mechanical waves during tissue expansion. Nat Phys 2012;8:628–34. 10.1038/nphys2355

[13] Lee KJ, Cox EC, Goldstein RE. Competing patterns of signaling activity in dictyostelium discoideum. Phys Rev Lett 1996;76:1174–7. 10.1103/PhysRevLett.76.1174 10061652

[14] Goldstein RE. Traveling-wave chemotaxis. Phys Rev Lett 1996;77:775–8. 10.1103/PhysRevLett.77.775 10062899

[15] Dormann D, Weijer CJ. Propagating chemoattractant waves coordinate periodic cell movement in Dictyostelium slugs. Development. 2001;128:4535–43. 11714678 10.1242/dev.128.22.4535

[16] Théry C, Ostrowski M, Segura E. Membrane vesicles as conveyors of immune responses. Nat Rev Immunol. 2009;9:581–93. 10.1038/nri2567 19498381

[17] Potolicchio I, Carven GJ, Xu X, Stipp C, Riese RJ, Stern LJ, et al Proteomic analysis of microglia-derived exosomes: metabolic role of the aminopeptidase CD13 in neuropeptide catabolism. J Immunol. 2013;175: 2237–43. 10.4049/jimmunol.175.4.2237 16081791

[18] Vincent C, Siddiqui TA, Schlichter LC. Podosomes in migrating microglia: components and matrix degradation. J Neuroinflammation. 2012;9:190 10.1186/1742-2094-9-190 22873355 PMC3423073

[19] Fetler L, Amigorena S. Brain under surveillance: the microglia patrol. Neuroscience. 2005;309:329–93. 10.1126/science.1114852 16020721

[20] Nasu-Tada K, Koizumi S, Inoue K. Involvement of *β*1 integrin in microglial chemotaxis and proliferation on fibronectin: Different regulations by ADP through PKA. Glia. 2005;52:98–107. 10.1002/glia.20224 15920726

[21] Yang TD, Kwon TG, Park J-S, Lee KJ. Trail networks formed by populations of immune cells. New J Phys. 2014;16:023017 10.1088/1367-2630/16/2/023017

[22] Honda S, Sasaki Y, Ohsawa K, Imai Y, Nakamura Y, Inoue K, Kohsaka S. Extracellular ATP or ADP induce chemotaxis of cultured microglia through Gi/o-coupled P2Y receptors. J Neurosci. 2001;21(6):1975–82. 11245682 10.1523/JNEUROSCI.21-06-01975.2001 PMC6762617

[23] Dou Y, Wu H-J, Li H-Q, Qin S, Wang Y-E, Li J, et al Microglial migration mediated by ATP-induced ATP release from lysosomes. Cell Research. 2012;22:1022–33. 10.1038/cr.2012.10 22231629 PMC3367529

[24] Lämmermann T, Afonso PV, Angermann BR, Wang JM, Kastenmüller W, Parent CA et al Neutrophil swarms require LTB4 and integrins at sites of cell death in vivo. Nature. 2013;498:371–5. 10.1038/nature12175 23708969 PMC3879961

[25] Friedl P, Wolf K. Tumour-cell invasion and migration: diversity and escape mechanisms. Nat Rev Cancer. 2003;3:362–74. 10.1038/nrc1075 12724734

[26] Satulovsky J, Lui R, Wang Y-L. Exploring the control circuit of cell migration by mathematical modeling. Biophys J. 2008;94:3671–83. 10.1529/biophysj.107.117002 18199677 PMC2292371

[27] Yang TD, Park J-S, Choi Y, Choi W, Ko T-W, Lee KJ. Zigzag Turning Preference of Freely Crawling Cells. Plos One. 2011;6(6):e20255 10.1371/journal.pone.0020255 21687729 PMC3110194

[28] Keller EF and Segel LA. Model for chemotaxis. J Theor Biol. 1971;30:225 10.1016/0022-5193(71)90050-6 4926701

