# Supplementary figures and images for: Enhancement of Chemotactic Cell Aggregation by Haptotactic Cell-To-Cell Interaction
Source: PLoS One. 2016 Apr 29;11(4):e0154717. doi: 10.1371/journal.pone.0154717 (PMC4851333; doi:10.1371/journal.pone.0154717)

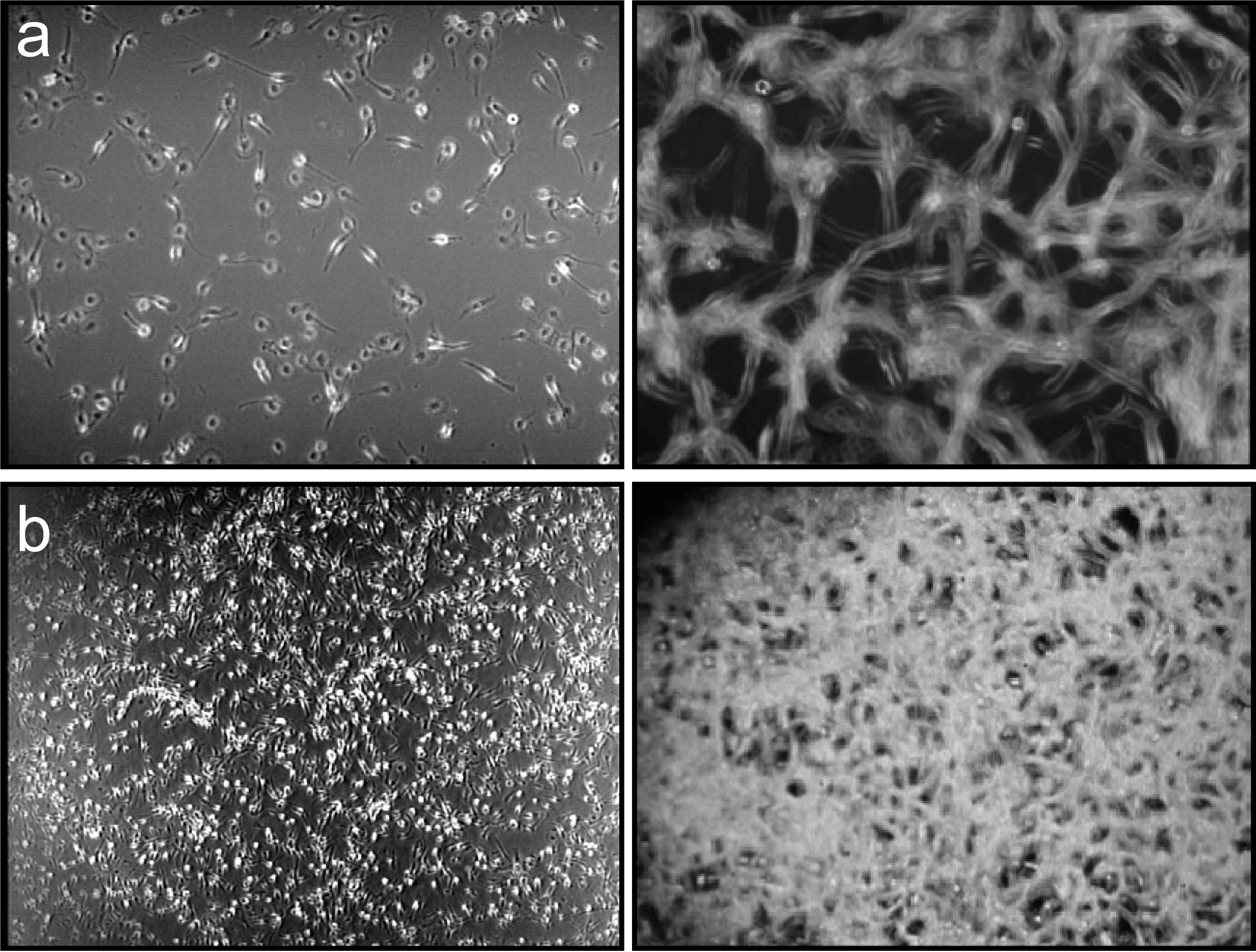

Supplement: S1 Fig — Cell density = 200 cells/mm2 in (a) and 1,000 cells/mm2 in (b). (50 hr duration of) 6,000 successive phase-contrast snapshot images taken at every 30 seconds (beginning from 3 days in vitro after the initial seeding) are superimposed to reveal the existence of trail patterns on the second column. (TIF) [file pone.0154717.s001.tif]

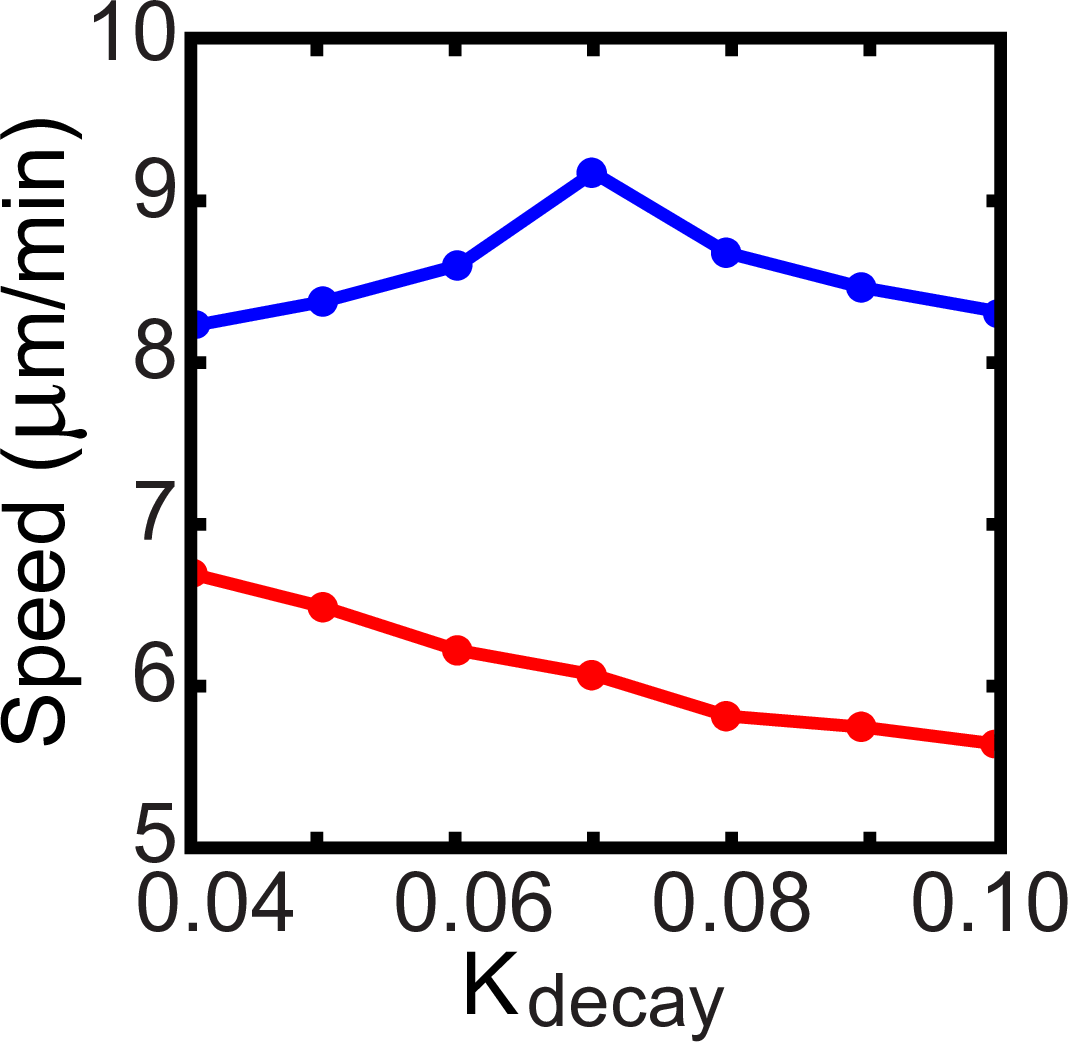

Supplement: S2 Fig — The speeds are estimated based on a time interval of 60 s. (TIF) [file pone.0154717.s002.tif]

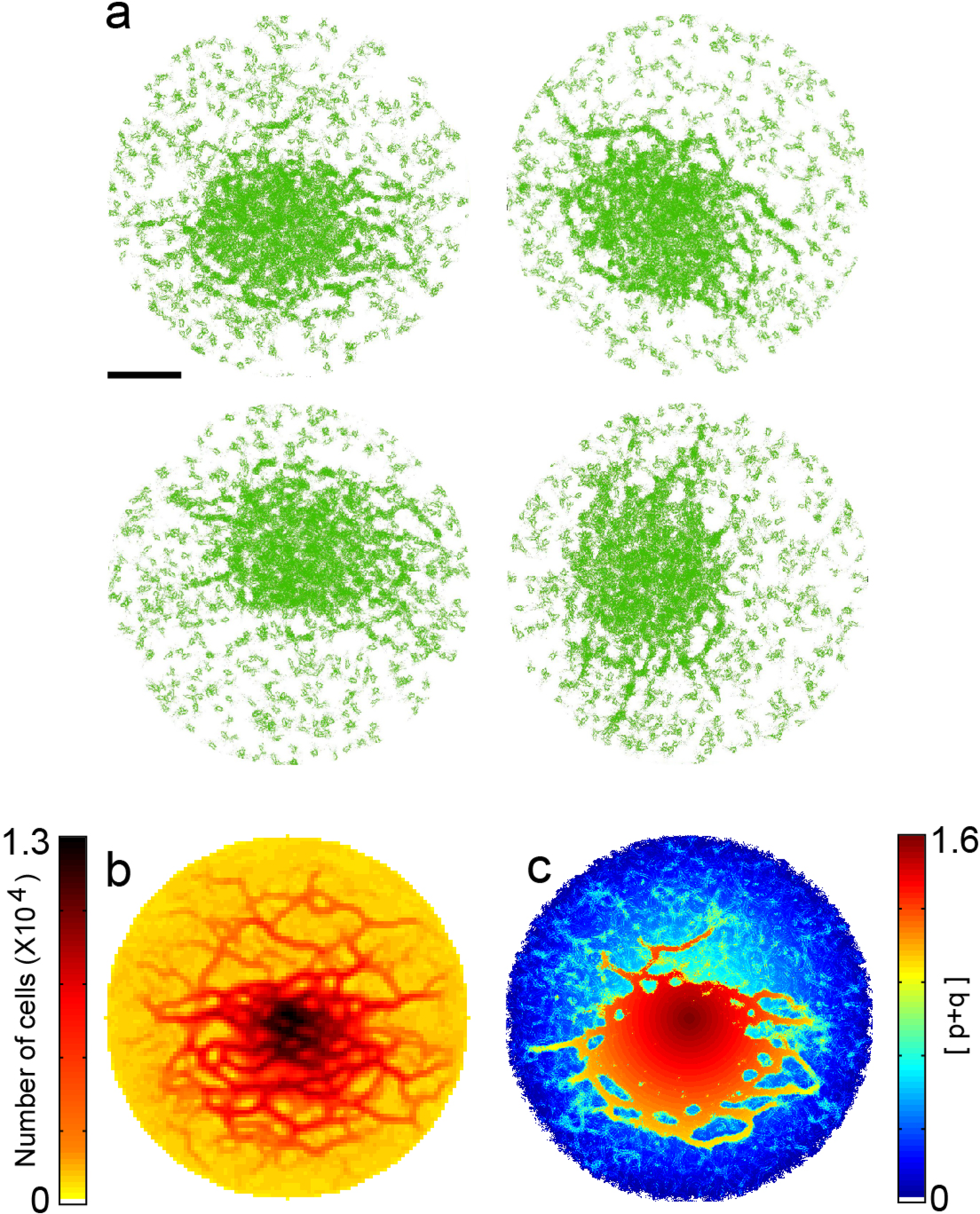

Supplement: S3 Fig — (a) Four different steady states for four different initial seeding conditions. (b) Time-accumulated (48 hr) cell count map of the case shown in the top-left frame in (a). (c) Snapshot image of p + q concentration map of the steady state shown in (b). The cell count map shown in (b) is based on a two-dimensional array of a small disk (of radius 10 pixels or 2.86 μm) windows. (TIF) [file pone.0154717.s003.tif]
